# Endosialin-positive tumor-derived pericytes promote tumor progression through impeding the infiltration of CD8^+^ T cells in clear cell renal cell carcinoma

**DOI:** 10.1007/s00262-023-03372-z

**Published:** 2023-01-16

**Authors:** Tong Lu, Jiayu Zhang, Shiqi Lu, Fa Yang, Lunbiao Gan, Xinjie Wu, Hongtao Song, Shaojie Liu, Chao Xu, Donghui Han, Bo Yang, Weihong Wen, Weijun Qin, Lijun Yang

**Affiliations:** 1 Xijing Hospital, Fourth Military Medical University, 127 Changle West Road, Xi’An, 710032 China; 2 Institute of Medical Research, Northwestern Polytechnical University, Xi’an, 710072 China

**Keywords:** Endosialin/CD248/TEM-1, Clear cell renal cell carcinoma, Tumor-derived pericyte, CD8^+^ T cell, ICB therapy

## Abstract

**Background:**

Immune checkpoint blockade (ICB) therapy can be effective against clear cell renal cell carcinoma (ccRCC), but many patients show no benefit. Tumor-derived pericytes (TDPs) may promote tumor progression by influencing T cells and are an immunotherapy target; however, they may comprise functionally distinct subtypes. We aimed to identify markers of tumor-promoting TDPs and develop TDP-targeting strategies to enhance ICB therapy effectiveness against ccRCC.

**Methods:**

We analyzed the relationship between endosialin (EN) expression and cytotoxic T-lymphocyte (CTL) infiltration in ccRCC tumor samples using flow cytometry and in a ccRCC-bearing mice inhibited for EN via knockout or antibody-mediated blockade. The function of EN^high^ TDPs in CTL infiltration and tumor progression was analyzed using RNA-sequencing (RNA-seq) data from ccRCC tissue-derived TDPs and single-cell RNA-seq (scRNA-seq) data from an online database. The role of EN in TDP proliferation and migration and in CTL infiltration was examined in vitro. Finally, we examined the anti-tumor effect of combined anti-EN and anti-programmed death 1 (PD-1) antibodies in ccRCC-bearing mice.

**Results:**

High EN expression was associated with low CTL infiltration in ccRCC tissues, and inhibition of EN significantly increased CTL infiltration in ccRCC-bearing mice. RNA-seq and scRNA-seq analyses indicated that high EN expression represented the TDP activation state. EN promoted TDP proliferation and migration and impeded CTL infiltration in vitro. Finally, combined treatment with anti-EN and anti-PD-1 antibodies synergistically enhanced anti-tumor efficacy.

**Conclusion:**

EN^high^ TDPs are in an activated state and inhibit CTL infiltration into ccRCC tissues. Combined treatment with anti-EN and anti-PD-1 antibodies may improve ICB therapy effectiveness against ccRCC.

**Supplementary Information:**

The online version contains supplementary material available at 10.1007/s00262-023-03372-z.

## Introduction

Renal cell carcinoma (RCC) accounts for 3–5% of all malignancies, with 75% being of the clear cell (ccRCC) type [1]. Immune checkpoint blockade (ICB) therapy, such as with antibodies against programmed death 1 (PD-1) and cytotoxic T lymphocyte antigen 4 (CTLA-4), has shown promising effectiveness in patients with ccRCC [2]. However, > 50% of patients show no benefit, and some patients who are initially sensitive to the therapy may develop secondary resistance [3, 4]. Therefore, new therapeutic targets are needed in ccRCC.

Tumor-derived pericytes (TDPs) are important cellular components of the tumor microenvironment (TME), with roles in tumor angiogenesis, metastasis, resistance to treatment, and patient mortality [5, 6]. Recently, TDPs were shown to be involved in cross-talk with tumor cells, endothelial cells, and immune cells in the TME, resulting in the promotion of tumor progression [7]. Several TDP-targeting strategies have been developed [8]; however, similar to cancer-associated fibroblasts (CAFs), TDPs are heterogeneous and may have different origins [9, 10]. Therefore, for precise therapeutic targeting, it is critical to both identify the tumor-promoting TDP subtypes and describe specific markers for these.

Endosialin (EN), also known as tumor endothelial marker 1 (TEM1) or CD248, is a transmembrane glycoprotein of the C-type lectin-like receptor family [11] and is highly expressed on CAFs and TDPs [12]. Previously, we found that EN^+^ CAFs promoted hepatocellular carcinoma progression by recruiting and inducing M2 polarization of macrophages [13]; however, the function of EN in TDPs has not yet been elucidated. EN has been shown to regulate platelet-derived growth factor-induced cell proliferation and migration in TDPs [14]; additionally, EN^+^ TDPs may promote tumor cell intravasation, thereby facilitating distant metastasis [15]; however, whether they promote tumor progression by other mechanisms, such as by influencing T cell infiltration, is unknown.

Here, we examined the relationship between EN expression and the infiltration of CD8^+^ T cells, or cytotoxic T lymphocytes (CTLs), in ccRCC clinical samples. We then examined the effect on CTL infiltration and tumor growth of EN inhibition via knockout or antibody-mediated blockade. We next examined whether EN^+^ TDPs could inhibit CTL infiltration in vitro. Additionally, using knockdown or antibody-mediated blockade, we examined the function of EN in TDP proliferation, migration, and tube formation. Finally, we investigated whether combined treatment with anti-EN and anti-PD-1 antibodies inhibited ccRCC growth in vivo.

## Materials and methods

### Culture of cell lines and isolation of primary stromal cells or pericytes

The human retinal pericyte cell line HRMVP and the mouse RCC cell line Renca were purchased from Aoyinbio Co., Ltd. (Shanghai, China), while the mouse prostate cancer (PCa) cell line RM1 and mouse melanoma cell line B16F10 were purchased from Zqxzbio Co., Ltd. (Shanghai, China). The cells were maintained in Dulbecco’s Modified Eagle’s Medium (DMEM) supplemented with 10% fetal bovine serum (FBS) (Thermo Fisher Scientific, Waltham, MA, USA; #A3161002C) and 1% penicillin–streptomycin (#15070063, Gibco). Primary stromal cells or pericytes were isolated from tumor samples obtained between May 2021 and June 2021 from patients with ccRCC or lung cancer at Xijing Hospital, Fourth Military Medical University (Xi'an, China), according to previously described protocols [6, 16].

### Flow cytometric analysis of ccRCC clinical samples

This study was approved by the Ethics Committee of Fourth Military Medical University. The ccRCC samples were obtained between February 2021 and June 2021 from 80 patients at Xijing Hospital, Fourth Military Medical University, with clinical information collected from electronic medical records. Type I and type IV collagenases were used to convert fresh ccRCC samples into single-cell suspensions. Cells were incubated in darkness for 30 min at 4 °C with one of three groups of antibodies and analyzed using a flow cytometer: Group 1 included antibodies against CD4, CD25, CD127, and FoxP3 (#A42924, Invitrogen); Group 2 included antibodies against CD3, CD4, CD8, CD279, CD366, and CD152 (Thermo Fisher Scientific; #11003742, #11004842, and #12008842; BioLegend, San Diego, CA, USA; #329916, #345014, and #369612); Group 3 included antibodies against CD326, CD31, CD45, and EN (Invitrogen; #17579182, #303105, #304029, and BioLegend, #949902). Prior to use, the anti-EN antibody IgG78 was conjugated with fluorescein using a kit (Frdbio, Wuhan, China; ARL0021K). TDPs were identified as the cells that remained after the exclusion of epithelial (CD326^+^), endothelial (CD31^+^), and immune cells (CD45^+^).

### Western blot

Protein samples was separated by 8% SDS-PAGE and transferred onto a polyvinylidene difluoride membrane (Thermo Fisher Scientific). The membrane was incubated with primary antibodies overnight at 4 °C, followed by incubation with horseradish peroxidase-conjugated secondary antibodies, and then visualized by chemiluminescence. The primary antibodies used were anti-EN (Abcam, Cambridge, UK; #ab48185), anti-GAPDH (Proteinintech, Rosemont, IL, USA; #10,494–1-AP), and anti-p-MAPK and anti-MAPK (Cell Signaling Technology, Danvers, MA, USA; #4370 T and #4695 T.

### Animal experiments

The animal experiments in this study were approved by the Laboratory Animal Welfare and Ethics Committee of Fourth Military Medical University. EN-knockout (EN^KO^) C57BL/6 mice were purchased from the Shanghai Model Organisms Center (Shanghai, China; #NM-KO-200094). BALB/c mice (6–8 weeks old, female, body weight 18–23 g) were purchased from the Animal Center of Fourth Military Medical University (Xi'an, China) and maintained in a 12-h light/12-h dark cycle with free access to food and water. Each mouse was subcutaneously inoculated in the back with 2 × 10^6^ tumor cells. For the antibody treatment, mice were inoculated with anti-mouse PD-1 antibody (50 mg/kg; Bio X Cell, Lebanon, NH, USA; #BE0033-2) with or without anti-EN antibody (5 mg/kg) every 4 d for a total of four times. Tumor size was measured every 4 d, tumor growth curves were drawn, and the survival of mice was observed. When the tumor diameter reached 2 cm or when severe weakness was observed, mice were euthanized and tumors isolated. The tumor samples were digested into a single-cell suspension, and associated T cells were analyzed using flow cytometry with fluorescently labeled anti-CD3, anti-CD4, anti-CD8, and anti-CD69 antibodies (#100306, #100412, and #100708, Biolegend).

### RNA-seq and scRNA-seq analysis

RNA was isolated from TDPs harvested from ccRCC samples from EN^high^ or EN^low^ groups and analyzed using the two-terminal sequencing model of the Illumina HiSeq sequencing platform (Genergy Bio-Technology, Shanghai, China). ScRNA-seq data for ccRCC were obtained from 48 samples from 38 patients [17–20].

ScRNA-seq dataset analysis was performed using the Seurat package (v4.0.5) in R (v4.1.0) [21]. First, individual gene expression matrices were used to create distinct Seurat objects. Second, strict quality-control procedures were performed: data were filtered to exclude cells that expressed < 200 genes, > 8,000 genes, > 20% mitochondrial genes, or > 0.1% hemoglobin genes; genes expressed in fewer than three cells were also excluded. Third, all Seurat objects were combined into one dataset, which was normalized and scaled using the NormalizeData and ScaleData functions, respectively. The FindVariableFeatures function was used to identify variable genes. Based on the variable genes, principal component analysis (PCA) was conducted using the RunPCA function. The datasets and corrected batch effects from different samples were integrated using the Harmony package (v0.1.0) [22]. Finally, clustering was conducted using the FindNeighbors and FindClusters functions with a resolution of 0.8. Visualization was implemented via t-distributed stochastic neighbor embedding.

Marker genes of cell clusters were identified using the FindAllMarkers function via the Wilcoxon rank-sum test. Each cell cluster was renamed as a specific cell type according to classical marker genes: B cells (*CD79A* and *MS4A1*), plasma cells (*CD79A*, *IGKC*, and *IGLC2*), CD4^+^ T cells (*CD3D*, *CD4*, and *IL7R*), CTLs (*CD3D*, *CD8A*, and *GZMB*), dendritic cells (*CD1C* and *CD1E*), endothelial cells (*PECAM1* and *vWF*), epithelial cells (*EPCAM* and *KRT18*), macrophages (*CD68* and *CD163*), mast cells (*CPA3* and *KIT*), monocytes (*CD14* and *S100A8*), natural killer cells (*NKG7* and *GNLY*), and stromal cells (*ACTA2* and *RGS5*).

### Immunohistochemistry (IHC)

IHC was performed using primary antibodies against human EN (#ab204914, Abcam) and CD8 (#70306S, CST). The expressions of EN and CD8 were assessed according to the percentage and intensity of the staining [23, 24].

### T cell infiltration assay

EN-silenced (si-EN) or control HRMVP cells (7.5 × 10^5^) were plated in pericyte medium on 8.0-mm-pore Matrigel-coated Transwell filters in the upper 24-mm chamber (Corning, Glendale, AZ, USA) and cultured for 24 h. Meanwhile, human peripheral blood mononuclear cells (PBMCs) were isolated from healthy donors, and T cells were isolated using the Dynabeads FlowComp human CD3 kit (#11365D, Invitrogen). T cells (5 × 10^5^) in DMEM supplemented with 5% fetal calf serum (FCS) were added to the upper chamber, while DMEM supplemented with 20% FCS was added to the lower chamber. After 24-h incubation, cells in the lower chamber were collected for flow cytometric analysis by using an anti-CD8 antibody (BioLegend; #344717).

### Cell proliferation and migration assay

Cell proliferation was measured using the cell-counting kit 8 (CCK8) assay (Mishubio, Xi’an, China). Si-EN or control cells were seeded into 96-well plates (3 × 10^3^ cells/well) and incubated for the indicated time. At the indicated time points, 10 μL of CCK8 reagent was added to each well followed by incubation at 37 °C for 1 h. Plates were then gently shaken in the dark for 5 min before absorbance at 450 nm was measured using a microplate reader.

To measure cell migration, a wound healing assay was performed. First, HRNVP cells were either transfected with siRNA or incubated with anti-EN antibody or control IgG for 45 min. After 48 h, cells were seeded into a six-well plate (5 × 10^5^ cells/well) and cultured for 12 h. Next, a scratch was made using a 200-μl pipette tip, and the culture medium was changed to serum-free medium. Images were taken (as time point 0 h) and the plates were returned to the incubator; further images were taken at 12 h, 24 h, and 48 h.

### Tube formation assay

Matrigel (BD Biosciences) was added to a 48-well plate (150 μL/well) and incubated at 37 °C for 30 min. Primary stromal cells were suspended by brief exposure to 0.25% trypsin (Invitrogen) and incubated with anti-EN antibody or control IgG in 50% phosphate-buffered saline (PBS)/45% basal media/5% FBS for 45 min, then seeded onto the Matrigel-covered plate. Si-EN and control HRNVP cells or primary pericytes in 300 μL of PBS/media/2% FBS were added to the Matrigel-covered plate (21,000 cells/well) and placed in a humidified 37 °C incubator for 16 h. Tubes/networks were imaged using Metamorph Image Analysis Software (Molecular Devices, Sunnyvale, CA).

### Statistical analysis

All statistical analyses were performed using IBM SPSS statistical (version 23) or GraphPad Prism 8 software. Results were presented as the mean ± SD. Comparisons were analyzed using two-tailed Student’s t test or one-way ANOVA followed by Dunnett’s post-hoc test. Differences were considered statistically significant at P < 0.05.

## Results

### High EN expression negatively correlates with CTL infiltration in patients with ccRCC

**Fig. 1:**
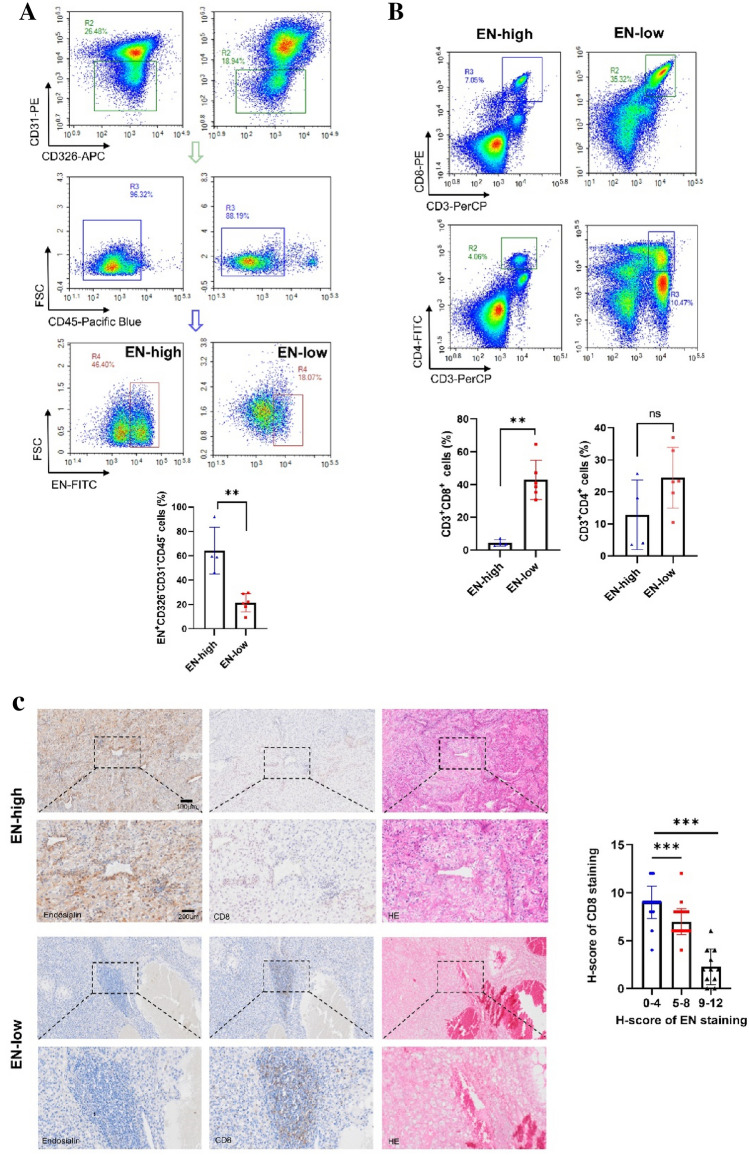
High EN expression negatively correlates with CTL infiltration in patients with ccRCC **A** Upper panel, flow cytometry analysis of human ccRCC tissues to show the percentage of intratumoral pericytes (CD326^−^CD45^−^CD31^−^) in EN-high and EN-low patients. Lower panel, quantification of the flow cytometry data (*n* = 10). **B** Upper panel, flow cytometry analysis of human ccRCC tissues to show the infiltrated CD8^+^ T cells (CD3^+^CD8^+^) and CD4^+^ T cells (CD3^+^CD4^+^) in EN-high and EN-low patients. Lower panel, quantification of the flow cytometry data (*n* = 10). **C** Left panel, IHC staining of endosialin and CD8 in EN-high and EN-low human ccRCC tissues. Right panel, quantitative analysis of the H-scores of EN and CD8 after IHC staining (*n* = 80). Representative data are shown. Data are presented as mean ± SD (**P < 0.01, ***P < 0.001)

Using the GEPIA database, we found a negative correlation between CD8A and endosialin expression (Supplementary Figure 1A). Therefore, we examined the percentage of EN^+^ TDPs in fresh nephrectomy samples from ten patients with ccRCC; the patients’ clinical information is shown in Supplementary Table 1. We chose to exclude epithelial cells, endothelial cells and immune cells, retaining as much TDPs as possible for detection. And through identification, we confirmed that these cells were TDPs (Supplementary Figure 2A-B). Results showed that the percentage of EN^+^ TDPs was 20–80% and, using a cut-off value of 40%, we divided patients into EN^high^ and EN^low^ groups (Fig. 1A). We analyzed the T cell infiltration in the samples and found that CTL infiltration was much lower in the EN^high^ group than in the EN^low^ group, while no significant difference was observed in CD4^+^ T cell infiltration (Fig. 1B). The expression of the T cell exhaustion markers PD-1, CTLA-4, and Tim-3 showed no significant difference between the two groups (Supplementary Fig. 1B–D). Additionally, no significant difference was observed in FoxP3^+^ regulatory T cell (Treg) infiltration (Supplementary Figure 1E–F). Using IHC on 80 ccRCC samples, we found that patients with high EN expression exhibited less CTL infiltration, indicating a negative correlation between EN expression and CTL infiltration (Fig. 1C).

### Antibody blockade of EN promotes CTL infiltration and inhibits RCC growth in vivo

**Fig. 2:**
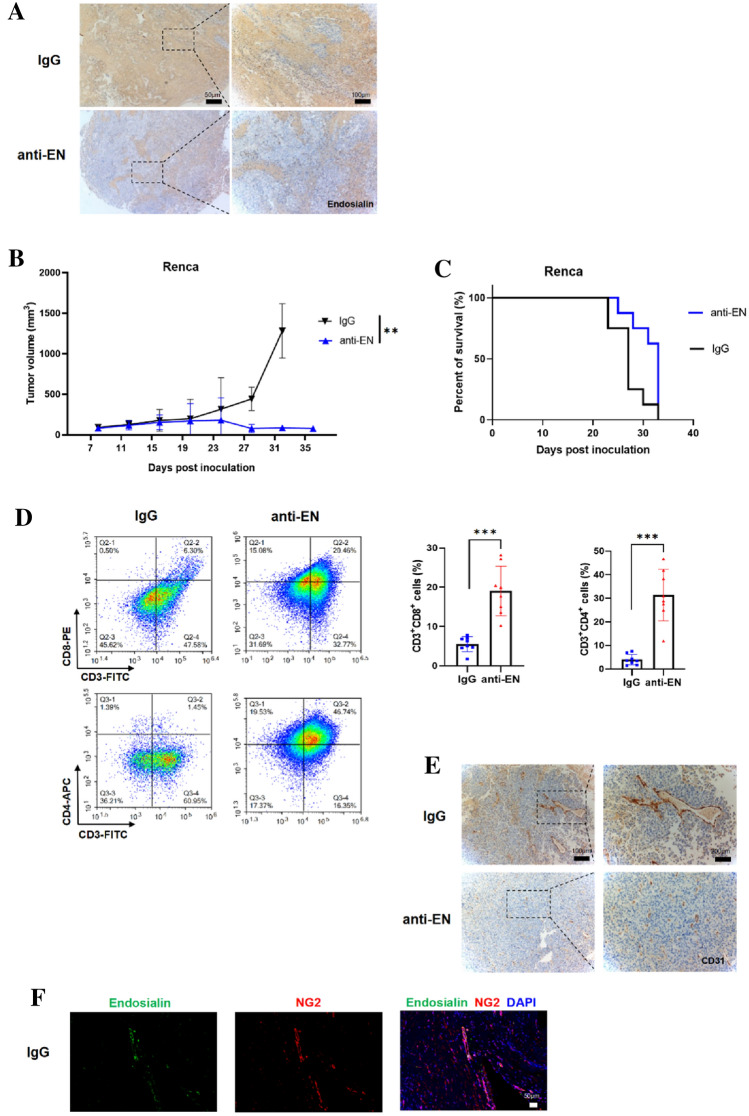
Antibody blockade of EN promotes CTL infiltration and inhibits RCC growth in vivo **A** IHC staining of endosialin in anti-EN and IgG groups. **B** Antibody blockade of endosialin inhibits tumor growth in RCC-bearing mice. **C** Antibody blockade of endosialin prolongs survival of RCC-bearing mice. **D** Left panel, flow cytometry of mouse RCC tissues to show the infiltration of CD8^+^ T cells (CD3^+^CD8^+^) and CD4^+^ T cells (CD3^+^CD4^+^) in anti-EN and control IgG-treated groups. Right panel, quantification of the flow cytometry data. **E** IHC staining of CD31 in anti-EN and IgG groups. **F** Immunofluorescence staining of endosialin and NG2 in IgG group. The number of samples in each group of all the above experiments was 8

To study the function of EN in CTL infiltration, we used anti-EN antibody in an RCC mouse model of BALB/c mice subcutaneously inoculated with Renca cells. EN expression significantly decreased in the anti-EN group (Fig. 2A), tumor growth significantly inhibited, and mouse survival increased (Fig. 2B–C). We next examined T cell infiltration in tumor tissues following anti-EN treatment and found that it increased for both CD8^+^ and CD4^+^ T cells compared to the control group (Fig. 2D).

We also found that blocking EN expression resulted in a significant decrease in mature blood vessels. Furthermore, we confirmed that EN was mainly expressed by pericytes in the RCC mouse model (Fig. 2E–F). These results indicate that EN inhibits T cell infiltration in RCC.

### ENKO mice exhibit increased CTL infiltration and impaired tumor growth

**Fig. 3:**
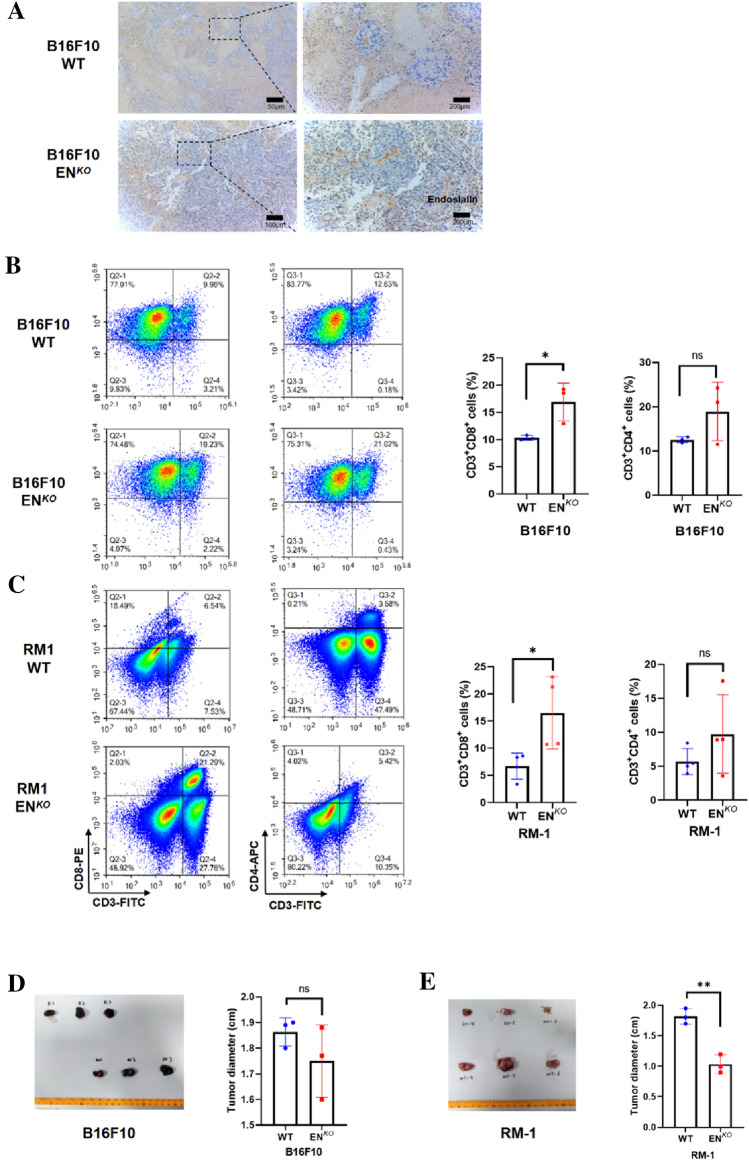
EN^KO^ mice exhibit increased CTL infiltration and impaired tumor growth **A** IHC staining of endosialin in EN^*KO*^ and WT mice (*n* = 3). **B** Left panel, flow cytometry analysis of melanoma tissues in EN^*KO*^ and WT mice to show the infiltration of CD8^+^ T cells (CD3^+^CD8^+^) and CD4^+^ T cells (CD3^+^CD4^+^). Right panel, quantification of the flow cytometry data (*n* = 3). **C** Left panel, flow cytometry analysis of PCa tissues in EN^*KO*^ and WT mice to show the infiltration of CD8^+^ T cells (CD3^+^CD8^+^) and CD4^+^ T cells (CD3^+^CD4^+^). Right panel, quantification of the flow cytometry data (*n* = 4). **D–E** Isolated tumor tissues and tumor volume analysis in EN^*KO*^ and WT mice (*n* = 3)

To determine the function of EN in CTL infiltration in tumors, we inoculated B16F10 and RM-1 cells into EN^KO^ or WT mice. We found that EN expression was lower in the EN^KO^ mice than in the WT mice (Fig. 3A). We also analyzed T cell infiltration in tumor tissues using flow cytometry. In both tumor models, results showed that CTL infiltration was significantly higher in the tumor samples of EN^KO^ mice than in WT mice; no significant differences were observed in CD4^+^ T cell infiltration (Fig. 3B–C). Additionally, tumor growth in both the melanoma and PCa cells was inhibited in EN^KO^ mice, consistent with the effect of the anti-EN antibody treatment (Fig. 3D–E). These results confirmed that EN impedes CTL infiltration and results in tumor progression.

### High EN expression represents the TDP activation state in ccRCC

**Fig. 4:**
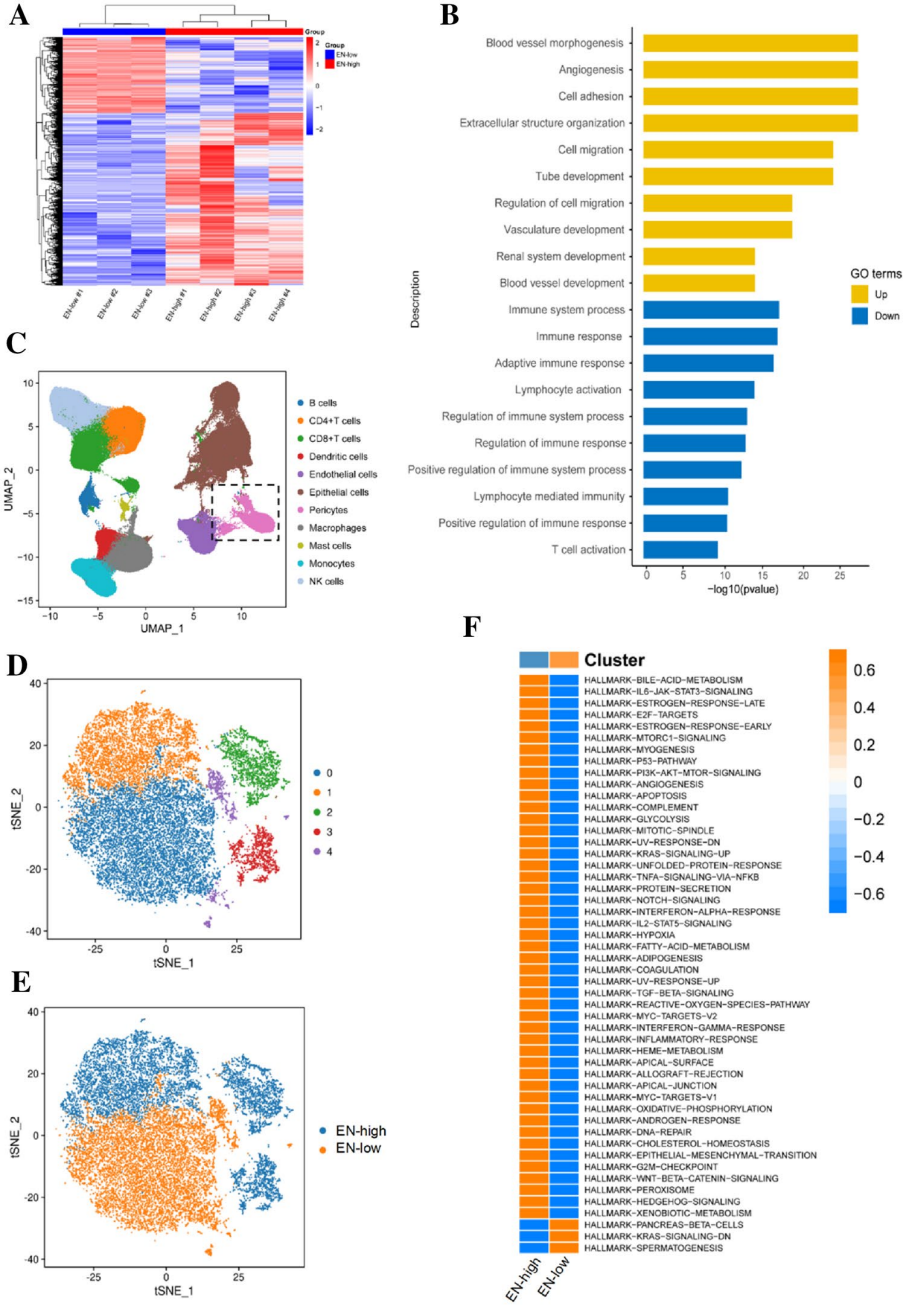
High EN expression represents the TDP activation state in ccRCC **A** Heatmap to show the different gene expression patterns in 4 EN-high and 3 EN-low ccRCC patients. **B** GO enrichment analysis to show that TDPs from EN-high patients had significantly increased gene expression that are related with the activation of TDPs. **C** U-MAP plot of integrated RCC transcriptomes to show the 11 cell types identified by graph-based clustering (*n* = 48). **D** T-SNE plot to show the five pericytes subclusters identified by graph-based clustering. **E** T-SNE plot to show the EN-high (blue) and EN-low (orange) pericytes. **F** Hallmarks of activated signaling pathways in EN-high and EN-low clusters

To determine how EN inhibits CTL infiltration, we analyzed TDPs isolated from the tumor tissues of four EN^high^ and three EN^low^ group members using RNA-seq (Fig. 4A). Gene Ontology enrichment analysis showed that highly expressed genes in TDPs from EN^high^ patients were related to blood vessel morphogenesis, angiogenesis, cell adhesion, extracellular structure organization, and cell migration, among others, thus indicating that the high EN expression related to TDP activation status (Fig. 4B). Due to the high heterogeneity of TDPs, which is difficult to overcome by RNA-seq, we integrated scRNA-seq data from 48 ccRCC samples and analyzed differentially expressed genes in EN^high^ and EN^low^ ccRCC tissues [17–20]. The results showed that ccRCC tumors contained a higher percentage of TDPs than adjacent normal tissues and that TDPs divided into five subclusters based on different expression signatures (Fig. 4C–D; Supplementary Figure 2A–B). Each subcluster had a unique expression signature, which reflected TDP heterogeneity (Supplementary Fig. 2C). Subclusters 1, 2, and 3 exhibited high EN expression and were proportionally increased in ccRCC tissues (Fig. 4E; Supplementary Figure 2D–E). In contrast to EN^low^ TDPs, EN^high^ TDPs exhibited activation in almost every signaling pathway, including those of p53, PI3K–Akt–mTOR, glycolysis, and transforming growth factor-beta (TGF-β) (Fig. 4F). These results suggest that high EN expression represents an activated state of TDPs in ccRCC.

### EN knockdown inhibits pericyte proliferation and migration and promotes CTL infiltration

**Fig. 5:**
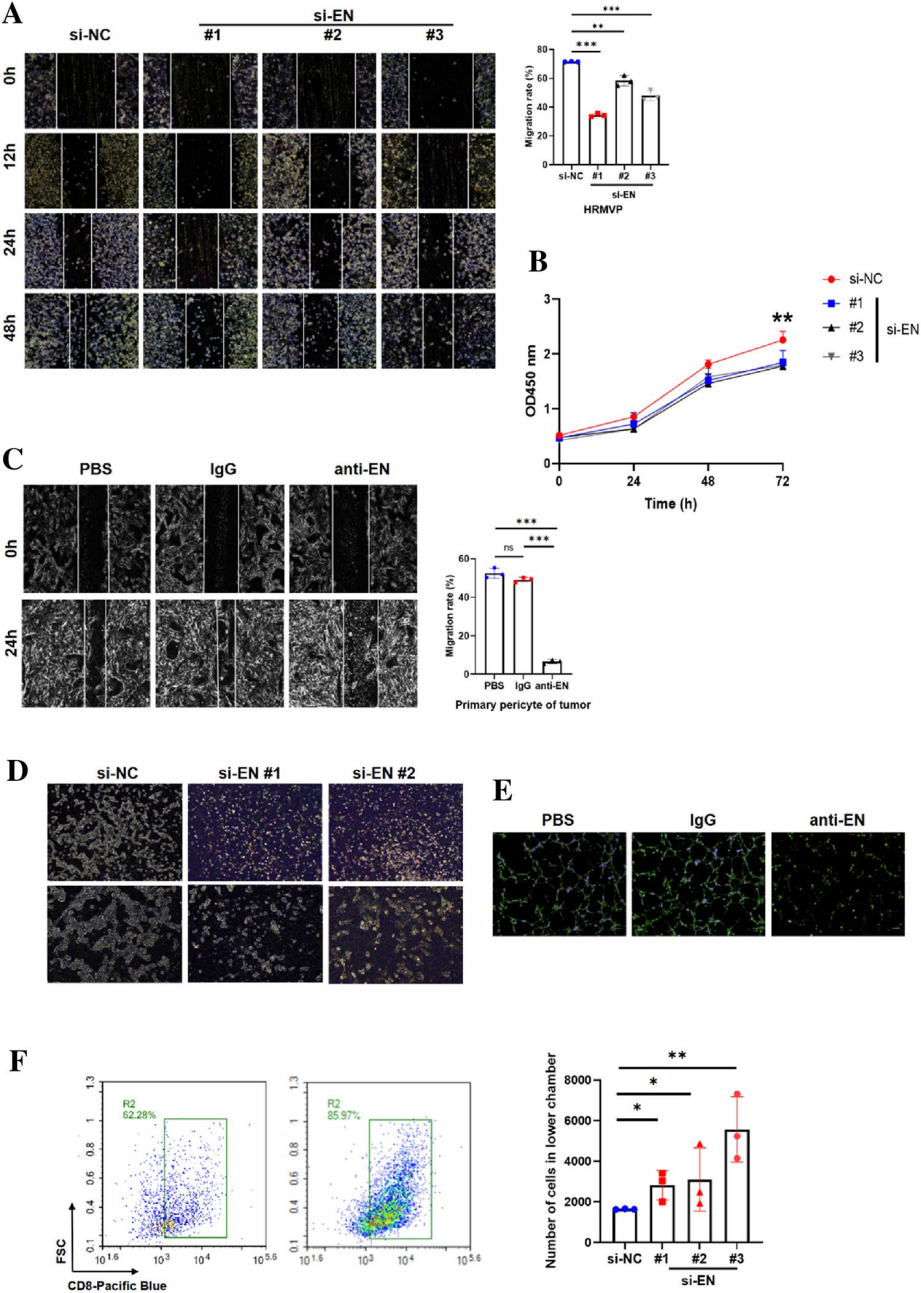
EN knockdown inhibits pericyte proliferation and migration and promotes CTL infiltration **A**Wound healing assay to show the inhibited migration of endosialin knockdown HRMVP cells. **B** CCK8 assay to show the inhibited proliferation of endosialin knockdown HRMVP cells (*n* = 4). **C** Wound healing assay to show the inhibited migration of primary pericytes after antibody blockade of endosialin. **D** Two-dimensional tube formation assay to show the inhibited tube formation of endosialin knockdown HRMVP cells. **E** Two-dimensional tube formation assay to show the inhibited tube formation of primary pericytes after antibody blockade. **F** Left panel, flow cytometry analysis to show the increased infiltration of T cells through the endosialin knockdown HRMVPs. Right panel, quantification of flow cytometry (*n* = 3)

Previous studies found that activated TDPs possess strong proliferative and migratory abilities [25, 26]. Based on our bioinformatic analysis, we speculated that EN may promote the activation of TDPs. Therefore, we examined pericyte proliferation and migration following EN knockdown or blockade by anti-EN antibody. Results showed that cell proliferation and migration were inhibited in EN^KO^ HRMVP cells (Fig. 5A–B). To corroborate this, we isolated TDPs from the fresh tumor tissue of a lung cancer patient and treated them with anti-EN antibody. The results showed that primary TDP migration was inhibited (Fig. 5C). We also examined the tube formation ability of HRMVP cells and primary TDPs after EN knockdown or anti-EN treatment and found that it was inhibited following both treatments (Fig. 5D–E). Additionally, we examined phosphorylation of ERK1/2 and Akt in EN^KO^ HRMVP cells and found that it was inhibited in both, indicating that the cells were inactive (Supplementary Fig. 3A). Using flow cytometry, we found no increased apoptosis in HRMVP cells following IgG78 treatment, ruling out toxicity of the antibody (Supplementary Fig. 3B). These results confirmed that EN is involved in maintaining the TDP activation state.

Since we previously found that EN may impede CTL infiltration into tumors, we examined whether EN^KO^ pericytes promote CTL infiltration by culturing EN^KO^ and control HRMVP cells in the upper Transwell chamber and analyzing the infiltrating T cells using flow cytometry. The results showed more infiltration through EN^KO^ HRMVP cells than through the control cells (Fig. 5F). In agreement with The Cancer Genome Atlas, we found that EN and CXCL12 expression positively correlated in EN^KO^ or blocked mice (Supplementary Fig. 3C–E). At high levels, CXCL12 (also called SDF1) acts as a chemorepellent of lymphocytes [27], which partly explains why EN impedes CTLs, though the mechanism remains unclear. Thus, we confirmed that EN inhibits CTL infiltration via pericytes.

### Combined anti-EN and anti-PD-1 antibody treatment inhibits RCC growth in vivo

**Fig. 6:**
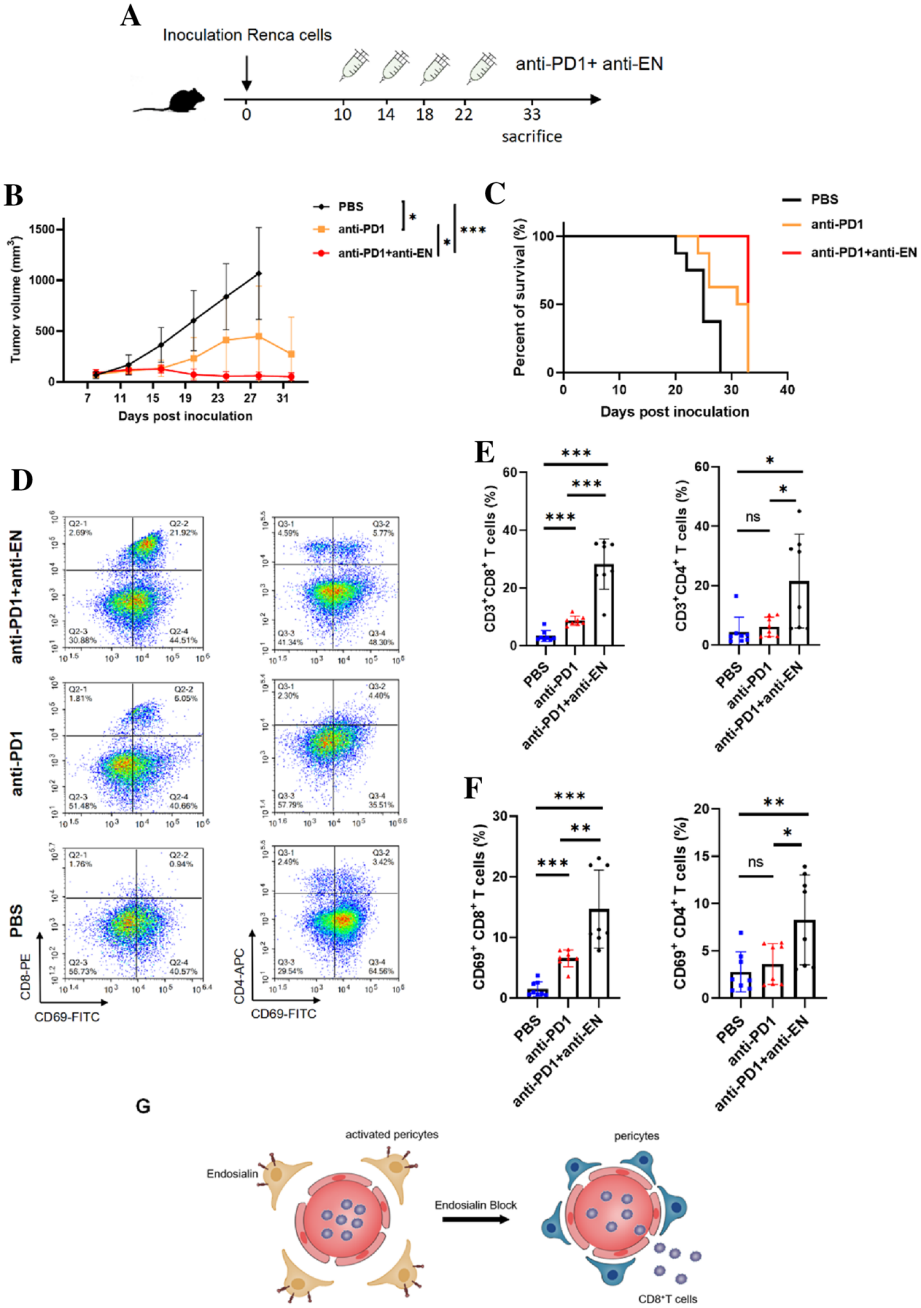
Combined anti-EN and anti-PD-1 antibody treatment inhibits RCC growth in vivo **A** Schematic image to show the process of combined treatment. Isolated RCC tissues after mice were sacrificed after treatment. **B** Tumor growth curves after treatment. **C** Survival curve of the RCC beating mice after treatment. **D** Flow cytometry analysis of intratumoral T cells in different groups after treatment. **E**–**F** Quantification of the flow cytometry data to show the intratumoral T cells (CD3^+^CD8^+^, CD3^+^CD4^+^) and active T cells (CD8^+^CD69^+^, CD4^+^CD69^+^) in different groups after treatment. **G** Graphic diagram to describe the function of endosialin, which inhibits the activation of TDPs and impede the infiltration of CD8^+^ T cells into tumor tissues. The number of samples in each group of all the above experiments was 8

Since EN blockade promoted CTL infiltration, we investigated whether combined anti-EN antibody and ICB treatment with anti-PD-1 could have a stronger anti-tumor effect in RCC-bearing mice (Fig. 6A). Compared to anti-PD-1 antibody alone, tumor growth was significantly inhibited and mice survived longer following combined treatment (Fig. 6B–C). Examining T cell infiltration and activation in tumor tissues using flow cytometry, we found that both CD4^+^ and CD8^+^ T cell infiltration increased following combined treatment and that infiltrating T cells were largely activated, as shown by increased percentages of both CD69^+^CD4^+^ and CD69^+^CD8^+^ T cells (Fig. 6D–F). These results indicate that combined treatment with anti-EN and anti-PD-1 antibodies can synergistically inhibit tumor growth and may overcome resistance to ccRCC ICB therapy.

## Discussion

Despite being immunogenic, RCC inhibits anti-tumor immunity, partially by upregulating immune checkpoint expression in the TME [3]. ICB is recommended as first-line therapy, and combination therapies involving ICB are now standard for patients with advanced RCC [28]. However, a substantial proportion of patients do not benefit from such approaches [4].

The factors that determine RCC responsiveness to ICB therapy have been subject to much research. Braun et al. found that CTL-infiltrated tumors have less PBRM1 mutations but are enriched for chromosomal losses of 9p21.3, explaining why PD-1 blockade is resisted in patients with high CTL infiltration [29]. Peng et al. reported that CTLs were proportionally reduced in recurrent RCC and were significantly associated with weaker immunotherapy responses [30]. Many combination therapies have been developed and applied in clinical trials to overcome ICB therapy resistance[31, 32]. Although these have dramatically improved the outcomes of patients with metastatic RCC, most patients either have primary resistance or acquire resistance to these therapies [4]. Identifying targets for RCC combination therapies is urgently required.

TDPs are important components of the TME, playing key roles in tumor progression. Recently, TDPs were found to regulate T cell function in the TME; e.g., glioma TDPs inhibited T cell proliferation by releasing prostaglandin E2, serum human leukocyte antigen G, hepatocyte growth factor, and TGF-β and were negatively associated with T cell infiltration [33]. Elsewhere, glioblastoma (GBM) TDPs strongly secreted anti-inflammatory cytokines and immunosuppressive molecules and have reduced co-stimulator expression, suppressing CD4^+^ T cell responses and IL-2 production in vitro [34]; additionally, GBM TDPs upregulated chaperone-mediated autophagy to enhance the expression of TGF-β and IL-10, which inhibit T cell function [35]. TDPs are, thus, an attractive target for immunotherapy [36].

TDPs are heterogeneous and may comprise functionally distinct subtypes. For precise targeting, it is important to identify specific markers of tumor-promoting TDPs. Regulator of G-protein signaling 5 (RGS5) inhibits TDPs maturation, and its knockout results in enhancing CTL infiltration and extending the survival of tumor-bearing mice [37]. Additionally, TDPs negatively influence CD4^+^ T cell activation and proliferation in an RGS5- and IL-6-dependent manner [25]. Other markers for tumor-promoting TDPs are still needed.

EN is expressed as a transmembrane glycoprotein specifically in CAFs and TDPs and is an immunotherapy target [38]. However, its function in TDPs requires elucidation. It might be required for TDP maturation and tumor vascularization, as EN^KO^ mice have more small vessels and less large vessels than WT mice [39]. Other tumor-promoting mechanisms of EN^+^ TDPs, such as through CTL infiltration, remain unknown.

Previously, we identified high EN expression in ccRCC which correlated with an immunosuppressive TME, such as increased Treg infiltration and immune checkpoints, and poor prognosis [40, 41]; however, the mechanism of this remained largely obscure. Here, we found that high EN expression correlated with low CTL infiltration in ccRCC tissues. In RCC-bearing mice, antibody blockade of EN promoted CTL infiltration and inhibited RCC growth. Analysis of both RNA-seq data of clinical ccRCC samples and scRNA-seq data of ccRCC revealed that EN^high^ TDPs represented an activated state. We then showed that EN knockdown or antibody blockade inhibited pericyte proliferation, migration, and tube formation ability and promoted CTL infiltration through pericytes in vitro. Finally, we showed that combined treatment with anti-EN and anti-PD-1 antibodies inhibited RCC growth in vivo.

In conclusion, our study demonstrated that high EN expression was exhibited in ccRCC and was representative of activated TDPs, which may inhibit CTL infiltration and thereby promote ccRCC progression. EN may provide a target for ccRCC treatment, and combined treatment of anti-EN and anti-PD-1 antibodies may enhance ICB therapy effectiveness in ccRCC.

## Supplementary Information

Below is the link to the electronic supplementary material.

Supplementary file1 (DOCX 1390 KB)

## Data Availability

The RNA-seq data from ccRCC patients are available in GEO database (https://www.ncbi.nlm.nih.gov/bioproject/PRJNA891024/). The scRNA-seq datasets generated and/or analyzed in this study are available in the Mendeley Data (https://doi.org/10.17632/nc9bc8dn4m.1); the Database of Genotypes and Phenotypes (phs002252.v1.p1; phs002065.v1.p1;) and the National Center for Biotechnology Information Gene Expression Omnibus (accession no.GSE159115).
