# Correction to: Endosialin‑positive tumor‑derived pericytes promote tumor progression through impeding the infiltration of CD8^+^ T cells in clear cell renal cell carcinoma

**DOI:** 10.1007/s00262-023-03427-1

**Published:** 2023-04-21

**Authors:** Tong Lu, Jiayu Zhang, Shiqi Lu, Fa Yang, Lunbiao Gan, Xinjie Wu, Hongtao Song, Shaojie Liu, Chao Xu, Donghui Han, Bo Yang, Weihong Wen, Weijun Qin, Lijun Yang

**Affiliations:** 1 Xijing Hospital, Fourth Military Medical University, 127 Changle West Road, Xi’an, 710032 China; 2 Institute of Medical Research, Northwestern Polytechnical University, Xi’an, 710072 China

**Correction to: Cancer Immunology, Immunotherapy** 10.1007/s00262-023-03372-z

The original version of this article unfortunately contained a mistake. The authors Weihong Wen and Weijun Qin should also have been denoted as a corresponding author.

