# Supplementary material for: Endosialin-positive tumor-derived pericytes promote tumor progression through impeding the infiltration of CD8+ T cells in clear cell renal cell carcinoma
Source: Cancer Immunol Immunother. 2023 Jan 16;72(6):1739–50. doi: 10.1007/s00262-023-03372-z (PMC10198862; doi:10.1007/s00262-023-03372-z)
Supplement: Supplementary file 1 — Supplementary file1 (DOCX 1390 KB) [file 262_2023_3372_MOESM1_ESM.docx]

**Supplementary figure and figure legends**

**
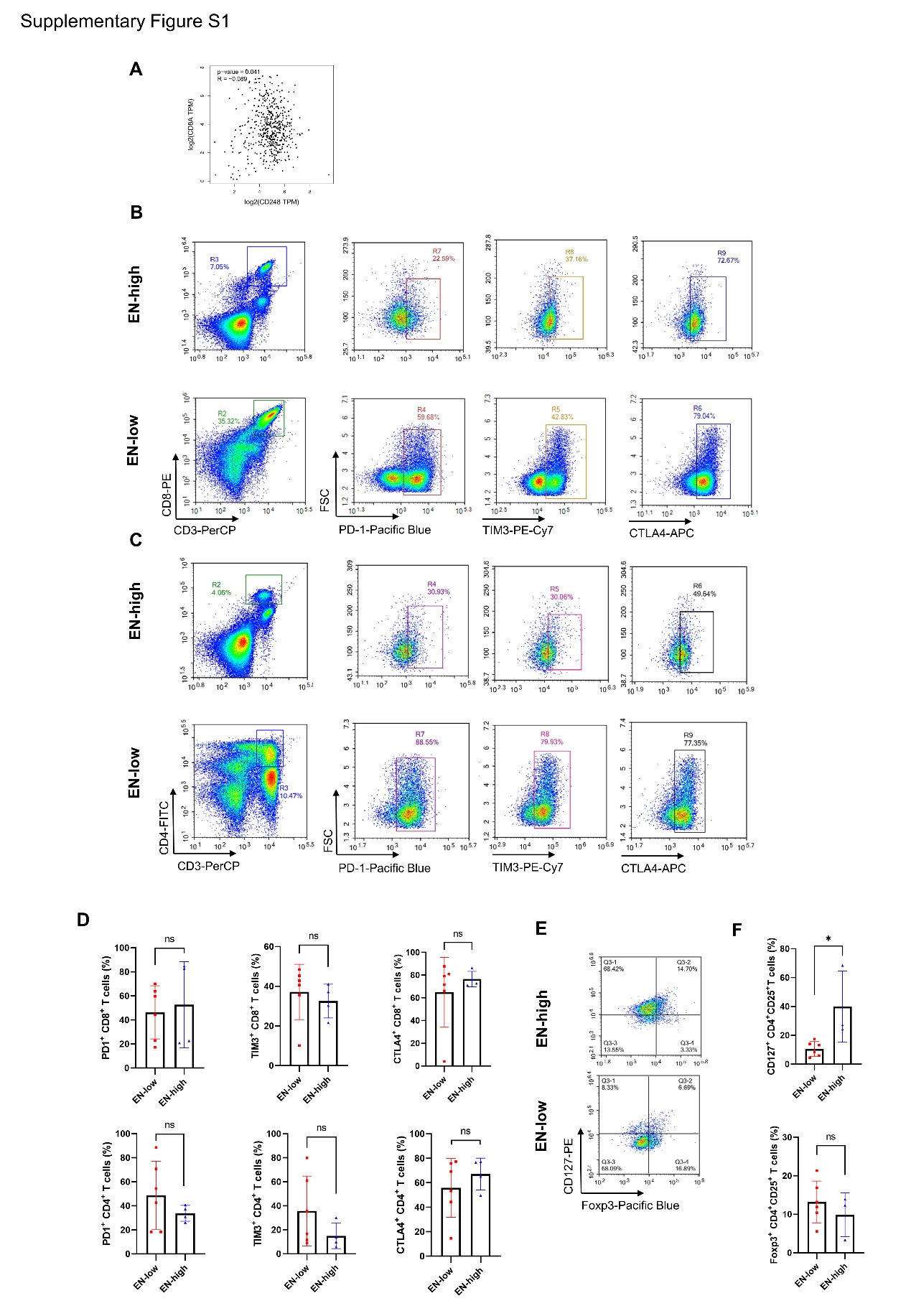
**

**Figure S1. Endosialin doesn’t impede Treg cells and exhausted T cells.**

(A) The negtive relationship between endosialin and CD8A in ccRCC patients which comes from GEPIA database. (B-C) Flow cytometry analysis to examine the expression of exhausted markers (PD-1, TIM-3 and CTLA4) in intratumoral CD8^+^ and CD4^+^ T cells in clinical ccRCC tissues. (D) Quantification of the flow cytometry data to examine intratumoral exhausted T cells (n=10). (E) Flow cytometry analysis to examine active Treg cells (CD4^+^CD25^+^Foxp3^+^) and inactive Treg cells (CD4^+^CD25^+^CD127^+^) in clinical ccRCC tissues. (F) Quantification of the flow cytometry data (n=10). Representative data are shown. Data are presented as mean ± SD (ns, no significant difference; *P < 0.05; **P < 0.01; ***P < 0.001).


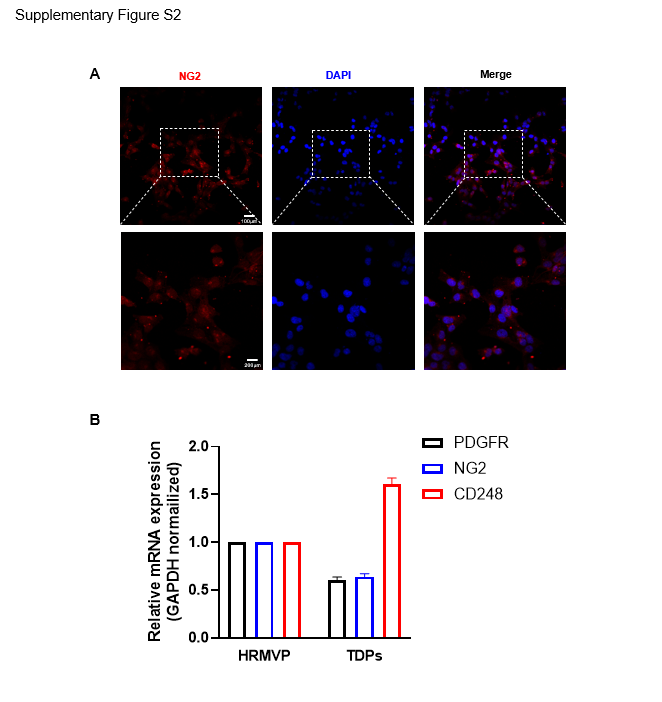


**Figure S2. Cell identification**

We chose to exclude epithelial cells, endothelial cells and immune cells, retaining as much TDPs as possible for detection. (A) Immunofluorescence showed that the sorted cells expressed NG2. (B) RT-PCR showed that the sorted cells expressed NG2, endosialin and PDGFR.


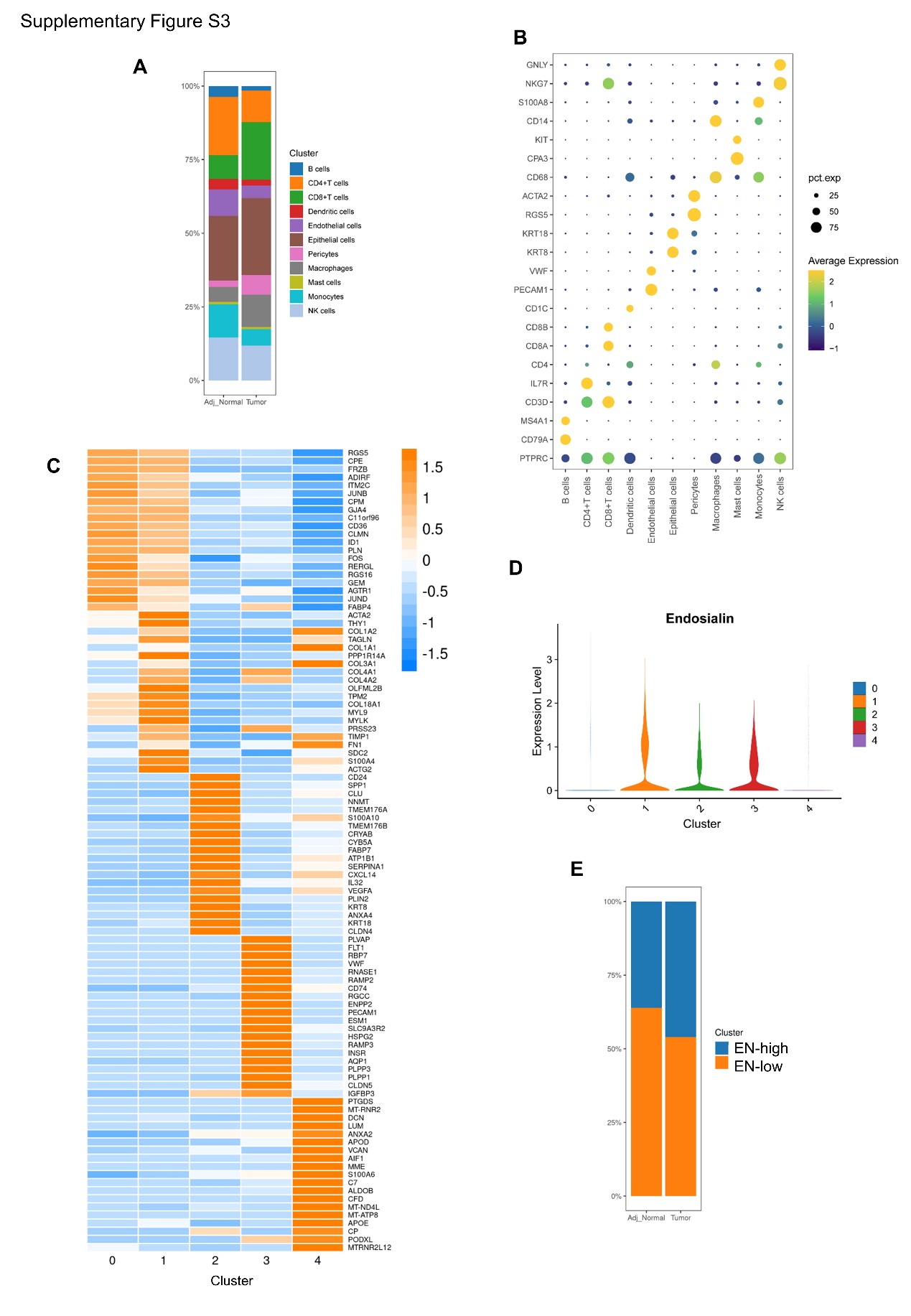


**Figure S3. TDPs can be divided into five different subclusters.**

(A) Bar graph to show the percentage of the cell clusters in RCC tissue and adjacent normal tissue. (B) Bubble chart to show the marker genes of each cell cluster. (C) Heatmap to show the hallmarks of five TDPs subclusters. Group 0 mainly expressed vascular related genes. Group 1 mainly expressed fibroblast related genes. Group 2 mainly expressed epithelial cell related genes. Group 3 mainly expressed endothelial cell related genes. Group 4 mainly expressed immunity related genes. (D) Violin plot to shows the expression of endosialin in five TDPs subclusters. (E) P bar graph to show the percentage of EN-high TDPs in RCC tissue and adjacent normal tissue.


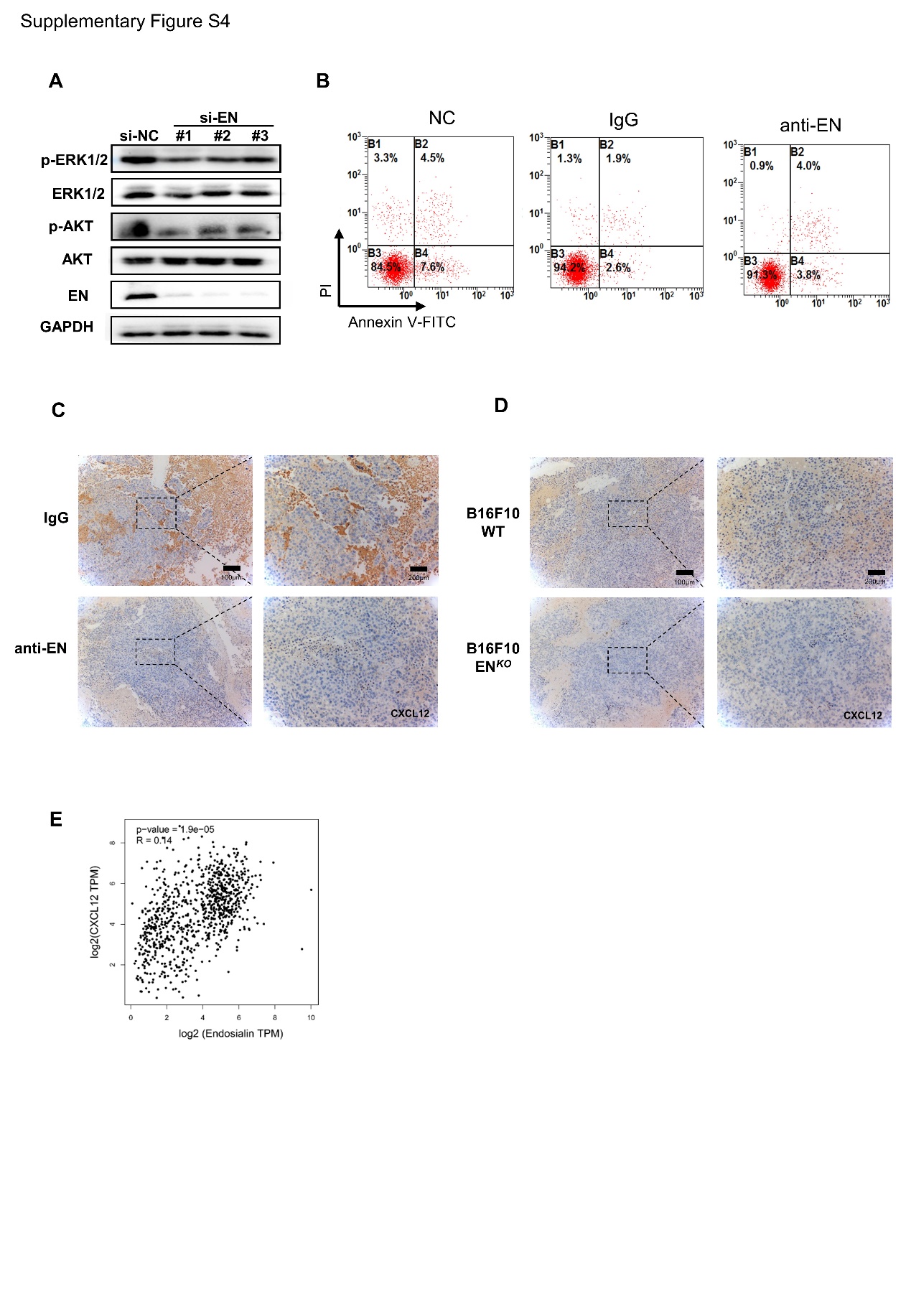


**Figure S4. The positive relationship between endosialin and CXCL12.**

(A) Western blot to show the activation of AKT and MAPK signal pathways in endosialin knockdown HRMVP cells. (B) HRMVP cells were incubated with IgG78 for 12 h before apoptosis analysis by flow cytometry after Annexin V/PI staining. (C) IHC staining of CXCL12 in anti-EN and IgG groups. (D) IHC staining of CXCL12 in EN*^KO^* and WT mice. (E) The positive relationship between endosialin and CXCL12 in RCC patients which comes from TCGA database.

**Supplementary Table 1: Demographic and Clinical Characteristics of the Patients at Baseline**

| Program | Number of Patients | Age | | Sex | | Visit Time | Tumor Type |
| --- | --- | --- | --- | --- | --- | --- | --- |
|  |  | range | average | male | female |  |  |
| fresh surgical specimens | 10 | 48-71 | 57 | 3 | 7 | 2021/5/20 to 2021/6/7 | ccRCC |
| Paraffin specimen | 80 | 13-86 | 55.47 | 47 | 33 | 2021/2/1 to 2021/6/30 | ccRCC |
| primary TDPs for RNA-seq | 7 | 25-65 | 48 | 5 | 2 | 2021/7/1 to 2021/7/12 | ccRCC |
| primary pericytes | 1 | 56 | 56 | 0 | 1 | 2021/9/10 | lung cancer |
